# Hydrogen bonding regulation-oriented design of pyridine sulfonate as a promising UV birefringent crystal characterized by enhanced structural anisotropy†

**DOI:** 10.1039/d4sc08583c

**Published:** 2025-02-14

**Authors:** Longyun Xu, Conggang Li, Shuaifeng Li, Huijian Zhao, Xianghao Kong, Zaixin Qu, Wenjie Feng, Kaidong Xu, Ning Ye, Zhanggui Hu

**Affiliations:** a School of Materials and Chemical Engineering, Henan University of Urban Construction Pingdingshan 467000 China; b Tianjin Key Laboratory of Functional Crystal Materials, Institute of Functional Crystal, Tianjin University of Technology Tianjin 300384 China cgli@email.tjut.edu.cn nye@email.tjut.edu.cn hu@mail.ipc.ac.cn; c State Key Laboratory of Crystal Materials, Shandong University Jinan 250100 China

## Abstract

Birefringent materials, capable of manipulating light polarization, are pivotal in advanced optical technologies. Traditionally, the development of such materials has predominantly focused on purely inorganic compounds, which often exhibit limited birefringence. Herein, we present a new 3-pyridinesulfonate birefringent crystal, Ca(3-C_5_H_4_NSO_3_)_2_·4H_2_O, synthesized *via* a hydrogen-bonded regulation strategy designed to enhance the coplanarity of [3-pySO_3_] groups. As expected, Ca(3-C_5_H_4_NSO_3_)_2_·4H_2_O demonstrates a notably large birefringence of 0.286@532 nm, exceeding that of most commercially available birefringent crystals. Furthermore, this compound demonstrates outstanding environmental stability and a short ultraviolet (UV) absorption cutoff edge at 257 nm, accompanied by a wide band gap of 4.4 eV. A combination of structural analysis and theoretical calculations unraveled the crucial role of hydrogen bonds in optimizing the arrangement of [3-pySO_3_] rings. This arrangement effectively induces a high degree of coplanarity and facilitates the formation of a quasi-2D layered structure, thereby contributing to the exceptional optical anisotropy of Ca(3-C_5_H_4_NSO_3_)_2_·4H_2_O. These findings highlight Ca(3-C_5_H_4_NSO_3_)_2_·4H_2_O as a promising UV birefringent crystal and underscore the efficacy of hydrogen bond engineering for designing new materials with enhanced birefringent properties.

## Abstract 2

A novel UV pyridine sulfate birefringent crystal, Ca(3-pySO_3_)_2_·4H_2_O, distinguished by large structural anisotropy, was prepared *via* a hydrogen bonding regulation strategy.

## Graphical abstract

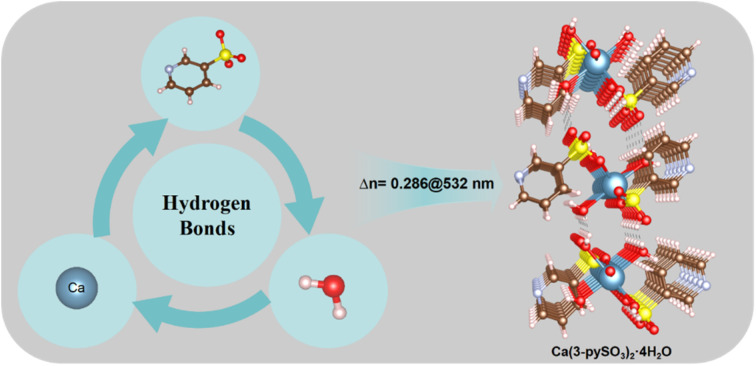

## 1. Introduction

Ultraviolet (UV) birefringent crystals are integral to the modulation of polarized light, with broad applications in optical communications, polarized light information processing, high-precision scientific instruments, and the laser industry.^1^ In the UV region (*λ* ≤ 400 nm), the performance limitations of commercially available birefringent crystals, such as α-BaB_2_O_4_ (α-BBO) and CaCO_3_, including phase transition instability and poor growth quality, highlight the urgent need to explore new crystals with significant birefringence in the UV window.^2–4^

Non-π-conjugated units, including (BO_4_)^5−^, (PO_4_)^3−^, and (SO_4_)^2−^, have garnered widespread attention due to their notably large HOMO–LUMO gaps and potential for applications within the short-wavelength range. Several crystalline materials containing (SO_4_)^2−^, such as NH_4_NaLi_2_(SO_4_)_2_ (0.009@546 nm), Cs_2_Mg_3_(SO_4_)_4_ (0.0016@534 nm), Cs_2_Ca_3_(SO_4_)_4_ (0.0043@534 nm) and Li_5_Cs(SO_4_)_3_ (0.0046@1064 nm), have been successfully developed.^5–8^ However, these materials exhibit relatively low birefringence (Δ*n* < 0.01@1064 nm), primarily due to their weak polarizability anisotropy and hyperpolarizability.^9,10^ In response to this situation, the introduction of heteroatoms or heterogeneous units into isotropic tetrahedra to generate anisotropic tetrahedra with enhanced polarizability anisotropy while maintaining a wide bandgap has attracted widespread interest.^5–7,11^ For example, newly designed hybrid tetrahedral units including [SO_3_F]^−^ and [SO_3_NH_2_]^−^ motifs have demonstrated increased polarizability anisotropy, functioning as birefringence-active functional building blocks (FBBs). In crystals such as Ba(SO_3_NH_2_)_2_ (0.03@1064 nm), KSO_3_F (0.019@546 nm), Ca(NH_2_SO_3_)_2_·H_2_O (0.033@1064 nm), Pb(NH_2_SO_3_)_2_·H_2_O (0.032@1064 nm), and Ba(SO_3_CH_3_)_2_ (0.04@589.3 nm), birefringence values exceeding 0.03@1064 nm have been observed, signifying a notable enlargement in optical anisotropy.^12–15^ Further enhancement of birefringence can be achieved by introducing π-conjugated groups into [SO_4_] units, which serve to increase both hyperpolarizability and optical anisotropy. A series of organic–inorganic hybrid 3-pyridinesulfonate crystals containing π-conjugated units, exemplified by A(3-pySO_3_)_2_·*x*H_2_O (A = Li, K, Rb, Ag, NH_4_, 3-pySO_3_ = 3-C_5_H_4_NSO_3_), were designed to exhibit a significant enhancement in birefringence at the wavelength of 589.3 nm, with values ranging from 0.240 to 0.312.^16^ The structural anisotropy is intricately linked to the geometric characteristics of the π-conjugated FBBs and their degree of coplanarity.^17^ Generally, a diminished dihedral angle between π-conjugated organic species corresponds to an increased birefringence.^18^

However, the usually large dihedral angle between pyridine rings in these materials often limits optical anisotropy.^16,19^ Recent research revealed that hydrogen bonds play an important role in regulating the spatial arrangement of organic fragments, thereby offering a way of regulating the structural anisotropy.^20^ For instance, the hydrogen bond formation between [NH_2_SO_3_] and [C_4_N_3_H_6_] results in the parallel alignment of [C_4_N_3_H_6_] groups.^21^ Likewise, the hydrogen bond interaction between [CF_3_SO_3_] and [CN_4_H_7_] units plays a crucial role in promoting the parallel orientation of [CN_4_H_7_] groups.^22^ Notably, the interaction between the planar hydrogen bond donor (C_5_H_6_ON)^+^ and the planar hydrogen bond acceptor (NO_3_)^−^ is pivotal in maintaining coplanarity throughout the crystal packing process observed in (C_5_H_6_ON)(NO_3_).^23^ Furthermore, the strategic incorporation of hydrogen bonds effectively optimizes the coplanarity of (H_2_C_6_N_7_O_3_) organic ligands, yielding an exceptionally large birefringence value of 0.60 observed in Cd(H_2_C_6_N_7_O_3_)_2_·8H_2_O.^24^

The pyridine ring contains large π-conjugated groups, functioning as an excellent chromophore and exhibiting both σ-donating and π-accepting properties, thereby positively influencing the optical characteristics of crystals.^25,26^ Alkaline-earth metals have a natural inclination to form hydrated [M(H_2_O)_*x*_] complexes, making them excellent hydrogen bond donors for controlling the arrangement of heterocyclic groups.^27^ Moreover, alkaline earth metal cations effectively inhibit d–d or f–f electronic transitions and widen the UV transparent window.^28^ Leveraging these properties, the integration of pyridine-based groups and alkaline-earth metal cations into the sulphate system provides a strategic approach for the assembly of hydrogen bonds, which optimizes the arrangement of π-conjugated groups and facilitates the fabrication of birefringent materials in the short-wave UV range. Inspired by these insights, a novel 3-pyridinesulfonate crystal, Ca(3-pySO_3_)_2_·4H_2_O (CPS), was rationally tailored by combining alkaline earth metal Ca^2+^ and [3-pySO_3_] FBBs with the donor–acceptor hydrogen bonding interactions. As expected, the construction of hydrogen bonds in this crystal effectively diminishes the dihedral angle between the pyridine rings. It is also found that hydrogen bonds play a crucial role in fostering the formation of quasi-two-dimensional (2D) layered structural networks and establishing an antiparallel symmetry of the distorted [CaO_7_] decahedral pseudo layers. These architectural factors significantly contribute to the increased birefringence of CPS. In this work, a series of measurements of CPS were conducted, including structural analysis, spectral analysis, environmental stability assessments, optical birefringence, and theoretical calculations.

## 2. Experimental section

### 2.1 Materials preparation

3-Pyridinesulfonic acid (3-C_5_H_5_NSO_3_, 98%) and calcium carbonate (CaCO_3_, 99%) were employed as raw materials without further purification. First, 3-C_5_H_5_NSO_3_ (1.5916 g, 10 mmol) and CaCO_3_ (0.4004 g, 4 mmol) were dissolved in deionized water under continuous stirring to obtain a clear solution. The solution was then left undisturbed under ambient conditions to facilitate crystal growth. After approximately two weeks, colourless single crystals of CPS were successfully obtained.

### 2.2 Crystal structure

Single-crystal X-ray diffraction measurements were performed using a Bruker SMART APEX III 4K CCD diffractometer with Mo Kα radiation (*λ* = 0.71073 Å) at 293(2) K. Absorption corrections were applied *via* the APEX IV software, using a multi-scan approach. The collected data were processed with the SAINT program and refined using Olex2, supplemented by SHEXT and SHEXL.^29^ Structural symmetry analysis was conducted with the PLATON program.^30^ Crystallographic information and refined structural data for CPS are summarized in Table S1,† while bond lengths, angles, and equivalent isotropic displacement parameters are detailed in Tables S2 and S3.†

### 2.3 Powder X-ray diffraction

Powder X-ray diffraction (PXRD) was conducted using a Rigaku SmartLab 9 kW diffractometer with monochromatized Cu Kα radiation (*λ* = 1.5418 Å). Measurements were carried out over a 2*θ* range of 10∼60°, with a step size of 0.01° and a step time of 2 seconds per step.

### 2.4 Stability assessment

The environmental stability of CPS samples was assessed *via* phase analysis after exposure to air and water atmospheres for durations of 0, 1, 3, 5, and 7 days, followed by PXRD characterization. Specifically, to evaluate stability in the water atmosphere, a 2 g sample of the title crystal powder was placed in a shallow plastic cap, which was then floated on the surface of 50 mL of deionized water contained in a Petri dish. Subsequently, the system was hermetically sealed to maintain a controlled environment, ensuring a reliable assessment of the crystal's stability when subjected to extended water exposure.^31^

### 2.5 Spectroscopy characterization

The ultraviolet-visible near-infrared (UV-vis-NIR) diffuse reflectance spectrum of CPS was recorded using a UH4150 spectrophotometer over the wavelength range of 200 to 1700 nm, with BaSO_4_ serving as the reference material. The optical band gap of CPS was determined using the Kubelka–Munk method.^32^ Additionally, the infrared (IR) spectrum of CPS was obtained with a Nicolet iS50 FT-IR spectrometer, covering the wavenumber range from 400 to 4000 cm^−1^.

### 2.6 Birefringence properties

Birefringence measurements were conducted using a Nikon Eclipse E200MV POL polarizing microscope equipped with a visible light source. The birefringence value was calculated using the following formula, *R* = Δ*n* × *T* = |*N*_e_ − *N*_o_| × *T*, where *R*, Δ*n*, *T*, *N*_e_, and *N*_o_ correspond to the optical path difference, birefringence, crystal thickness, ordinary wave birefringence, and extraordinary wave birefringence, respectively.^33,34^

### 2.7 Structure–property relationship

The total density of states (TDOS), partial density of states (PDOS), and band structure were analysed using the plane-wave pseudopotential method within the framework of density functional theory (DFT).^35–37^ The exchange–correlation potential was handled with the Perdew–Burke–Ernzerhof (PBE) functional within the generalized gradient approximation (GGA).^38,39^ A standard norm-conserving pseudopotential with a kinetic energy cutoff of 810 eV was utilized to ensure computational accuracy. A Monkhorst–Pack *k*-point grid of 4 × 3 × 2 was used for sampling and numerical integration in the Brillouin zone. Theoretical refractive indices and birefringence were derived based on electronic transitions.

## 3. Results and discussion

### 3.1 Synthesis and stability analysis

**Fig. 1:**
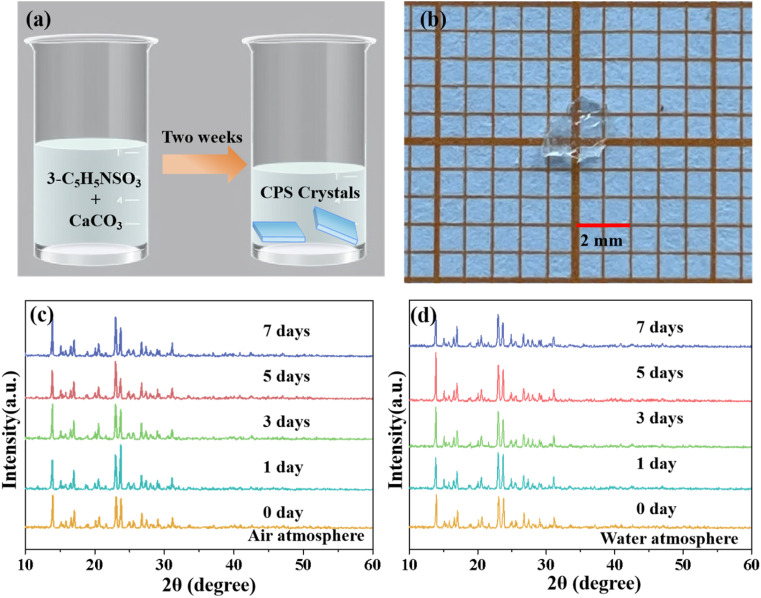
(a) Schematic diagram of crystal growth by the solvent evaporation method. (b) Transparent and millimetre-scale CPS crystal. (c and d) The PXRD patterns of CPS crystals under air and water atmosphere conditions over 7 days, respectively.

A colorless CPS single crystal approximately 2 mm in length was successfully grown using a solvent evaporation method over a period of approximately two weeks, as depicted in Fig. 1a and b. PXRD curves (Fig. S1†) confirm the high purity of CPS, with experimental data aligning closely with the calculated structural patterns. The environmental stability investigation of the CPS crystal mainly included air and water atmosphere stability. The environmental stability was characterized by the phase analysis of CPS samples exposed to air and water atmospheres for a duration of 1, 3, 5, and 7 days, using PXRD measurements. As illustrated in Fig. 1c and d, all the PXRD peaks of the samples exposed to the two atmospheres up to 7 days are still in accordance with those of the initial specimen, manifesting high atmospheric stability of CPS crystals.

### 3.2 Crystal structure of CPS

**Fig. 2:**
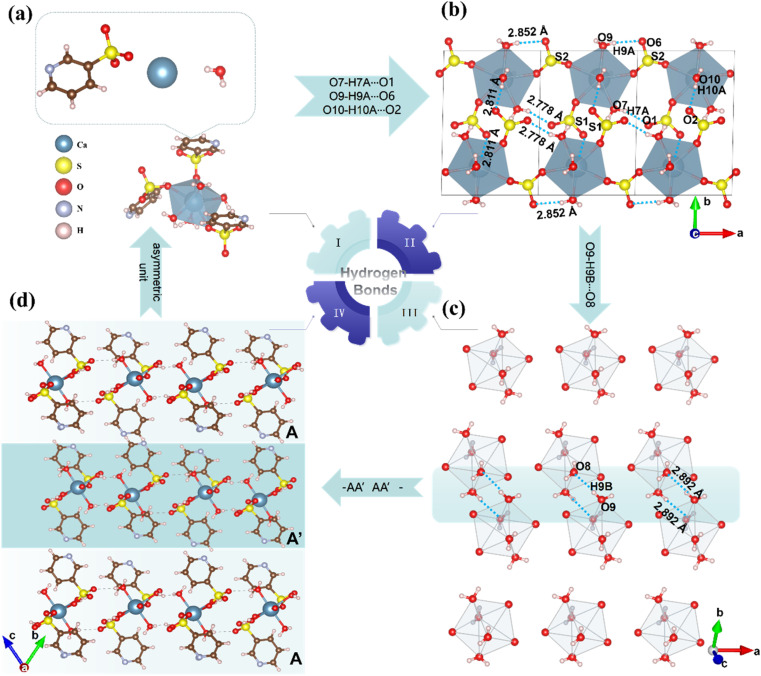
Structural characterization of CPS. (a) [3-pySO_3_] and [Ca(3-pySO_3_)_3_(H_2_O)_4_] motifs. (b) 1D hydrogen-bonded chain formed by O9–H9A⋯O6, O7–H7A⋯O1, and O10–H10A⋯O2. (c) Hydrogen bond network connected by O9–H9B⋯O8. (d) Quasi-2D layered crystal with an –AA′AA′– anti-parallel packing mode.

Single-crystal X-ray diffraction analysis revealed that CPS crystallizes in the centrosymmetric space group *P*1̄ (No. 2), with cell parameters *a* = 6.986(3) Å, *b* = 11.274(5) Å, *c* = 11.590(5) Å, and *V* = 843.3(6) Å^3^. The asymmetric unit comprises one distinct Ca atom, two [3-pySO_3_] groups, and four H_2_O molecules. As depicted in Fig. 2a, each Ca^2+^ cation coordinates with three [3-pySO_3_] groups and four H_2_O molecules, forming the basic structural unit [Ca(3-pySO_3_)_3_(H_2_O)_4_] with a distorted decahedron [CaO_7_]. Variations in bond lengths (2.307–2.496 Å) and bond angles (71.09–172.96°) contribute to this distortion (Table S2†). Fig. S2† illustrates the crystal structure, showcasing four distinct types of hydrogen bonds. The first two types are the O10–H10A⋯O2 and O7–H7A⋯O1 hydrogen bonds, in which [3-pyS1O_3_] groups act as hydrogen bond acceptors and H_2_O serves as the donor. The third type involves the O9–H9A⋯O6 hydrogen bond, with [3-pyS2O_3_] groups functioning as the acceptor and H_2_O as the donor. The fourth type is the O9–H9B⋯O8 hydrogen bond, which occurs between water molecules. The unit cell contains two basic structural units, [Ca(3-pySO_3_)_3_(H_2_O)_4_], interconnected by O10–H10A⋯O2 hydrogen bonds (2.811 Å). These units form a one-dimensional (1D) [Ca_2_(3-pySO_3_)_4_·8H_2_O]_∞_ chain, linked through O9–H9A⋯O6 hydrogen bonds (2.852 Å), O7–H7A⋯O1 hydrogen bonds (2.778 Å), and [SO_3_], as shown in Fig. 2b. The 1D chains further assemble into 2D [Ca_4_(3-pySO_3_)_8_ · 16H_2_O]_∞_ layers *via* O9–H9B⋯O8 hydrogen bonds (2.892 Å), as depicted in Fig. 2c. Since the hydrogen bond lengths range from 2.778 to 2.892 Å (Table S4†), they are generally consistent with those reported in the literature, and the corresponding interactions are relatively strong.^40^ Adjacent 2D layers are further stacked in an anti-parallel –AA′AA′– arrangement, forming the final quasi-2D layered structure (Fig. 2d). Among the reported pyridine sulfonates, Cs(3-pySO_3_) also adopts a 2D layered structure.^16^ However, in contrast to hydrogen-bonded frameworks, its 2D architecture is directly interconnected through –Cs–O–Cs– bonds. Hydrogen bonds, on the other hand, play a significant role in regulating the angle between π-conjugated organic groups with ring structures, often promoting their coplanar alignment.^2,24,41^ As depicted in Fig. S2,† the CPS crystal structure features two types of coplanar pyridine rings, corresponding to the pyridine groups in [3-pyS1O_3_] and [3-pyS2O_3_]. Hydrogen bonds play a critical role by stabilizing and orienting the [SO_3_] units in [3-pySO_3_] groups, indirectly influencing the alignment of the pyridine rings and enhancing their coplanarity. Notably, The dihedral angle between the two types of pyridine rings in CPS is 38.14° (Fig. 5a), which is not only smaller than the corresponding angle of 41.37° observed in Cs(3-pySO_3_) crystals but also less than those found in other 1D and 3D pyridine sulfonate crystals that lack hydrogen bonding interactions.^16^

### 3.3 Optical properties

**Fig. 3:**
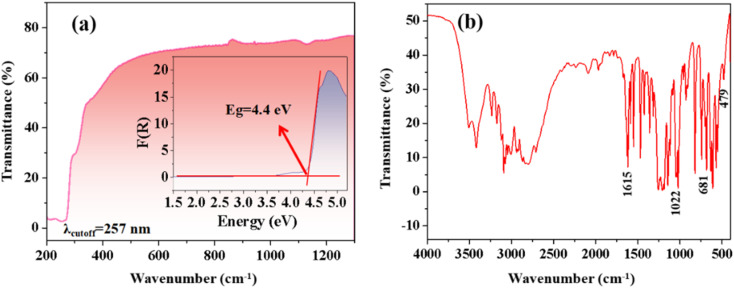
(a) UV-vis-NIR diffuse reflectance spectrum and the corresponding band gap for CPS. (b) Infrared spectrum of CPS.

The UV-vis-NIR diffuse reflectance spectrum of CPS, shown in Fig. 3a, reveals a short UV absorption cutoff edge of 257 nm. The experimental band gap value of 4.4 eV is determined by the Kubelka–Munk formula, as demonstrated in Fig. 3a.^32^ As indicated in Table S5,† CPS crystals exhibit a short cutoff edge in the UV region, akin to other sulfate materials. Notably, the cutoff edge of CPS crystals is comparatively shorter than that observed in the majority of pyridine sulfonate crystal derivatives, such as Ag(3-pySO_3_) (*λ*_cutoff_ = 325 nm), Rb(3-pySO_3_) (*λ*_cutoff_ = 289 nm), NH_4_(3-pySO_3_) (*λ*_cutoff_ = 283 nm), Cs(3-pySO_3_) (*λ*_cutoff_ = 283 nm), and Li(3-pySO_3_) (*λ*_cutoff_ = 275 nm).^16^ This finding highlights the shorter UV cutoff edge of CPS, underlining its potential as a UV birefringent material candidate. Additionally, the IR spectrum of CPS, shown in Fig. 3b, provides insight into its molecular vibrations. The broad peak near 3550 cm^−1^ and the strong absorption band at 1615 cm^−1^ are attributed to the stretching and bending vibrations of O–H bonds in crystalline water. The stretching vibrations of S–O bonds are identified between 1200 and 1000 cm^−1^. The bands at 1022 cm^−1^ and 681 cm^−1^ correspond to the breathing mode of the pyridine ring and out-of-plane deformation, respectively.^42,43^ The out-of-plane CH deformation vibrations occur within the range of 1010–820 cm^−1^, while peaks below 500 cm^−1^ are associated with Ca–O bond stretching vibrations.^14,43^

### 3.4 Birefringence characterization

**Fig. 4:**
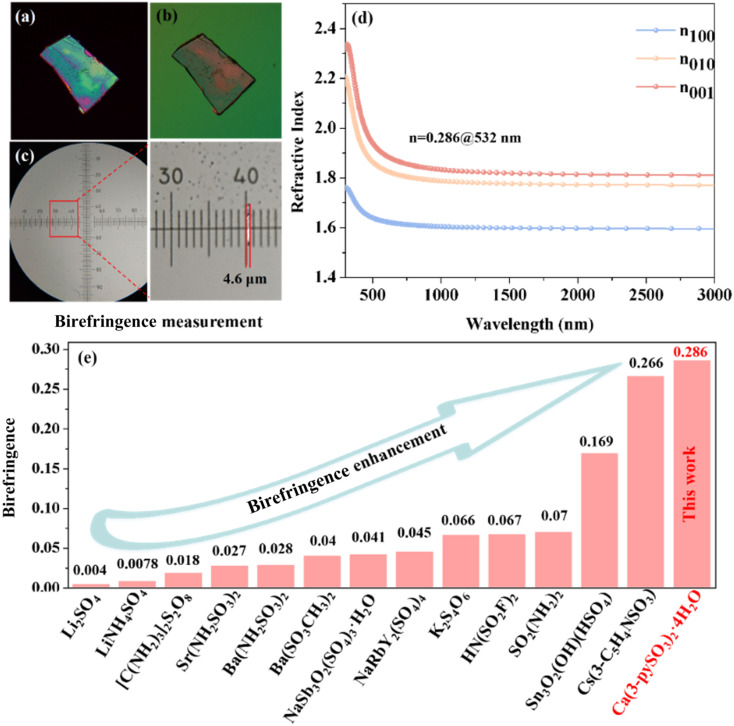
(a and b) The original interference color of the CPS crystal under cross-polarized light and an image of the CPS crystal after extinction. (c) Thickness of the measured CPS crystal. (d) The theoretical refractive index dispersion curve and birefringence of CPS. (e) The comparison of the birefringence among selected sulfate-related materials.

**Fig. 5:**
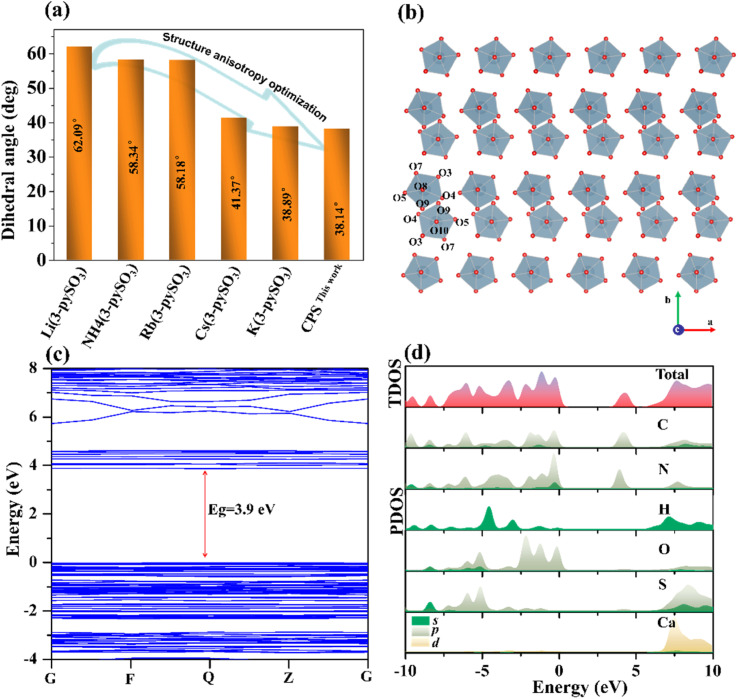
(a) A comparison of dihedral angles between pyridine rings in 3-pyridinesulfonate-type crystals. (b) The pseudo-layered structure built by antiparallel [CaO_7_] polyhedra in CPS. (c) Band structure for CPS. (d) TDOS and PDOS for CPS.

The birefringence of CPS was measured using a cross-polarization microscope. As shown in Fig. 4a and b, the original interference color of CPS was identified as fourth-order green under cross-polarized light. The measured thickness of the CPS crystal sample is approximately 4.6 μm (Fig. 4c). Experimental measurements determined the birefringence of CPS to be 0.29 in the visible region. Additionally, first-principles calculations, as shown in Fig. 4d, indicate a theoretical birefringence value of 0.286 at 532 nm, closely matching the experimental result. To the best of our knowledge, the experimentally determined birefringence of CPS exceeds that of the majority of sulfate-based materials, as demonstrated in Table S5† and Fig. 4e. This enhanced birefringence is also superior to that of other commercially available birefringent materials, such as LiNbO_3_ (0.074@546 nm), α-BaB_2_O_4_ (0.122@546 nm), YVO_4_ (0.21@633 nm), MgF_2_ (0.012@532 nm) and CaCO_3_ (0.172@532 nm).^33,44,45^ This enhanced birefringence observed in CPS can be attributed to the regulatory effects of hydrogen bonds in the crystal structure, which is manifested in two key aspects: (1) the reduced dihedral angles of the π-conjugated pyridine rings, which increase electronic polarization, along with the quasi-2D layered structure and distorted [CaO_7_] decahedron, exert a synergistic action in enhancing optical anisotropy;^46^ and (2) the formation of antiparallel [CaO_7_] decahedral pseudo-layers in CPS (Fig. 5b) further amplifies the birefringence of the CPS crystal.^47^

### 3.5 Structure–property relationship

To gain insight into the potential relationship between microstructure and optical properties, the electronic structure of CPS was investigated using density functional theory (DFT). As illustrated in Fig. 5c, CPS is identified as a direct bandgap compound with a theoretical optical bandgap value of 3.9 eV, slightly lower than the experimentally determined value of 4.4 eV. This difference mainly stems from the underestimation of the band gap by GGA technology. The TDOS and PDOS reveal the interrelationships between atomic orbitals and the origin of optical properties. The extended electronic structure of CPS was therefore calculated, as shown in Fig. 5d. The PDOS results show that the valence states (−2.6–0 eV) near the forbidden band are mainly contributed by C 2p, N 2p, and O 2p orbitals, while the bottom of the conduction states (3.5–5.1 eV) are primarily composed of C 2p and N 2p orbitals. In contrast, the bands situated between 7 and 10 eV, far from the forbidden band, are predominantly occupied by H 1s and Ca 4d orbitals. Notably, the O 2p orbital in the valence region is primarily influenced by the induction effect of the sulfonic groups on the pyridine ring. Consequently, these results indicate that the [3-pySO_3_] organic ligands are the key structural components responsible for the favorable optical anisotropy observed in CPS.

## 4. Conclusions

In summary, a novel alkaline earth metal organic–inorganic hybrid birefringent crystal, Ca(3-C_5_H_4_NSO_3_)_2_·4H_2_O, was successfully synthesized using a hydrogen bonding regulation approach. By controlling the [3-pySO_3_] organic ligand functional groups through hydrogen bonding interactions, a smaller dihedral angle between pyridine rings was achieved. This unique configuration further facilitated the formation of a quasi-2D layered structure and the antiparallel symmetry of the distorted [CaO_7_] polyhedra. Collectively, these factors contribute to the remarkably enhanced birefringence of 0.286@532 nm observed in Ca(3-C_5_H_4_NSO_3_)_2_·4H_2_O, surpassing that of most inorganic available birefringent crystals. Moreover, the title crystal exhibited excellent transmittance in the UV region, with a UV cutoff edge at 257 nm and a wide band gap of 4.4 eV. Additionally, stability analyses revealed that Ca(3-C_5_H_4_NSO_3_)_2_·4H_2_O displayed remarkable environmental stability. These findings highlight CPS as a promising birefringent crystal and present a novel approach for the design of high-performance crystals with large optical anisotropy.

## Data availability

The data supporting this article have been included as part of the ESI.† Crystallographic data for 2407675 have been deposited at the CCDC.

## Author contributions

Longyun Xu: experiments, investigation, data curation, writing the original draft. Conggang Li: conceptualization, funding acquisition, methodology, project administration, review & editing. Shuaifeng Li, Huijian Zhao, and Xianghao Kong: experiments, software, formal analysis. Zaixin Qu, Wenjie Feng, and Kaidong Xu: experiments. Ning Ye and Zhanggui Hu: resources, funding acquisition.

## Conflicts of interest

There are no conflicts to declare.

## Supplementary Material

SC-OLF-D4SC08583C-s001

SC-OLF-D4SC08583C-s002
