# Supplementary material for: Hydrogen bonding regulation-oriented design of pyridine sulfonate as a promising UV birefringent crystal characterized by enhanced structural anisotropy
Source: Chem Sci. 2025 Feb 14;16(12):5186–93. doi: 10.1039/d4sc08583c (PMC11840952; doi:10.1039/d4sc08583c)
Supplement: SC-016-D4SC08583C-s001 [file SC-016-D4SC08583C-s001.pdf]

Supplementary Information (SI) for Chemical Science.
This journal is © The Royal Society of Chemistry 2025

Supporting Information

Hydrogen bonding regulation-oriented design of pyridine sulfonate as a promising UV birefringent crystal characterized by enhanced structural anisotropy

Longyun Xu,[a,b] Conggang Li,*[,b,c] Shuaifeng Li,[b] Huijian Zhao,[b] Xianghao Kong,[b] Zaixin Qu,[a] Wenjie Feng,[a] Kaidong Xu,[a] Ning Ye,*[,b] and Zhanggui Hu*[,b]

[a]School of Materials and Chemical Engineering, Henan University of Urban Construction, Pingdingshan, 467000, China

[b]Tianjin Key Laboratory of Functional Crystal Materials, Institute of Functional Crystals, Materials Science and Engineering, Tianjin University of Technology, Tianjin 300384, China

[c]State Key Laboratory of Crystal Materials, Shandong University, Jinan, 250100, China

Corresponding Author **E-mail: cgli@email.tjut.edu.cn; nye@email.tjut.edu.cn; hu@mail.ipc.ac.cn*

1. **Table S1.** Crystal data and structural refinement for CPS.

2. **Table S2.** Selected bond lengths (Å) and angles (deg.) for CPS.

3. **Table S3.** Atomic coordinates (× 10^4) and equivalent isotropic displacement parameters (Å^2 × 10^3) for CPS. U(eq) is defined as one-third of the trace of the orthogonalized U_{ij} tensor.

4. **Table S4.** The related bond length (Å) and angle (°) of hydrogen bonds in CPS.

5. **Table S5.** Comparison of optical properties between selected sulfate-related materials.

6. **Fig. S1** Experimental and calculated PXRD patterns of CPS.

7. **Fig. S2** Types of hydrogen bonds in the structure of CPS and the coplanarity of pyridine rings in [3-pySO_3] groups.

Table S1. Crystal data and structural refinement for CPS.

empirical formula	$C_{10}H_{16}CaN_2O_{10}S_2$
formula weight	428.45
temperature (K)	273(2) K
wavelength (Å)	0.71073 Å
crystal system	triclinic
space group	$P\bar{1}$
a (Å)	6.986(3)
b (Å)	11.274(5)
c (Å)	11.590(5)
V (Å^3)	843.3(6)
Z	2
ρ_{caled} (g/cm^3)	1.687
F (000)	444
crystal size (mm^3)	0.02 × 0.06 × 0.07
R (int)	0.0668
completeness	100.0 %
GOF (F^2)	1.01
final *R* indices [$F_o^2 > 2\sigma(F_o^2)$]^a	R_1 = 0.0396, wR_2 = 0.0775
R indices (all data)	R_1 = 0.0729, wR_2 = 0.0895
CCDC number	2407675

$^aR_1 = \sum||F_o|-|Fc||/\sum|F_o|$; $wR_2 = [\sum w(F_o^2 - F_c^2)^2/\sum w(F_o^2)^2]^{1/2}$.

Table S2. Selected bond lengths (Å) and angles (deg.) for CPS.

Ca(1)-O(3)	2.393(2)	N(4)-C(5)	1.336(4)
Ca(1)-O(4)	2.496(2)	C(6)-C(7)	1.380(4)
Ca(1)-O(5)#1	2.485(2)	C(6)-H(6)	0.9300
Ca(1)-O(7)	2.395(2)	C(10)-C(9)	1.382(4)
Ca(1)-O(8)	2.395(2)	C(10)-H(10)	0.9300
Ca(1)-O(9)	2.412(2)	C(3)-C(4)	1.379(4)
Ca(1)-O(10)	2.307(2)	C(3)-C(2)	1.377(4)
S(1)-O(1)	1.442(2)	C(3)-H(3)	0.9300
S(1)-O(2)	1.456(2)	C(9)-C(8)	1.385(4)
S(1)-O(3)	1.4543(19)	C(7)-C(8)	1.381(4)
S(1)-C(9)	1.778(3)	C(7)-H(7)	0.9300
S(2)-O(4)	1.4567(19)	C(1)-C(2)	1.373(4)
S(2)-O(5)	1.4504(18)	C(1)-H(1)	0.9300
S(2)-O(6)	1.4618(19)	C(4)-C(5)	1.385(4)
S(2)-C(4)	1.772(3)	C(5)-H(5)	0.9300
N(1)-C(6)	1.337(4)	C(8)-H(8)	0.9300
N(1)-C(10)	1.335(4)	C(2)-H(2)	0.9300
N(4)-C(1)	1.337(4)		
O(3)-Ca(1)-O(4)	71.09(7)	O(6)-S(2)-C(4)	106.22(12)
O(3)-Ca(1)-O(5)#1	144.71(7)	O(3)-S(1)-O(2)	112.55(12)
O(3)-Ca(1)-O(8)	98.00(7)	O(3)-S(1)-C(9)	105.17(12)
O(3)-Ca(1)-O(9)	140.49(7)	O(2)-S(1)-C(9)	105.36(12)
O(3)-Ca(1)-O(7)	71.65(7)	O(1)-S(1)-O(3)	112.75(12)
O(5)#1-Ca(1)-O(4)	143.11(6)	O(1)-S(1)-O(2)	113.60(12)
O(8)-Ca(1)-O(4)	83.95(7)	O(1)-S(1)-C(9)	106.56(12)
O(8)-Ca(1)-O(5)#1	82.08(7)	S(1)-O(3)-Ca(1)	147.63(12)
O(8)-Ca(1)-O(9)	90.62(7)	S(2)-O(4)-Ca(1)	140.13(11)

O(8)-Ca(1)-O(7)	88.83(8)	S(2)-O(5)-Ca(1)#2	143.42(11)
O(10)-Ca(1)-O(3)	88.32(8)	C(5)-N(4)-C(1)	116.5(2)
O(10)-Ca(1)-O(4)	101.13(8)	C10-N(1)-C(6)	117.4(2)
O(10)-Ca(1)-O(5)#1	90.97(7)	N(1)-C(6)-C(7)	122.9(3)
O(10)-Ca(1)-O(8)	172.96(8)	N(1)-C(10)-C(9)	123.4(3)
O(10)-Ca(1)-O(9)	86.41(8)	C(2)-C(3)-C(4)	117.7(3)
O(10)-Ca(1)-O(7)	90.23(9)	C(10)-C(9)-S(1)	120.4(2)
O(9)-Ca(1)-O(4)	71.63(7)	C(10)-C(9)-C(8)	118.7(3)
O(9)-Ca(1)-O(5)#1	74.56(7)	C(8)-C(9)-S(1)	120.9(2)
O(7)-Ca(1)-O(4)	140.55(7)	C(6)-C(7)-C(8)	119.3(3)
O(7)-Ca(1)-O(5)#1	73.07(7)	N(4)-C(1)-C(2)	123.4(3)
O(7)-Ca(1)-O(9)	147.39(7)	C(3)-C(4)-S(2)	121.2(2)
O(4)-S(2)-O(6)	111.56(11)	C(3)-C(4)-C(5)	119.0(2)
O(4)-S(2)-C(4)	106.07(11)	C(5)-C(4)-S(2)	119.8(2)
O(5)-S(2)-O(4)	113.25(11)	N(4)-C(5)-C(4)	123.6(3)
O(5)-S(2)-O(6)	112.70(11)	C(7)-C(8)-C(9)	118.2(3)
O(5)-S(2)-C(4)	106.40(11)	C(1)-C(2)-C(3)	119.8(3)
Ca(01)-O(8)-H(8A)	109.6	N(1)-C(10)-H(10)	118.3
Ca(01)-O(8)-H(8B)	111(2)	C(9)-C(10)-H(10)	118.3
H(8A)-O(8)-H(8B)	111.3	C(4)-C(3)-H(3)	121.2
Ca(01)-O(10)-H(10A)	116(3)	C(2)-C(3)-H(3)	121.2
Ca(01)-O(10)-H(10B)	130(2)	C(6)-C(7)-H(7)	120.3
H(10A)-O(10)-H(10B)	111(4)	C(8)-C(7)-H(7)	120.3
Ca(01)-O(9)-H(9A)	109.7	N(4)-C(1)-H(1)	118.3
Ca(01)-O(9)-H(9B)	109.7	C(2)-C(1)-H(1)	118.3
H(9A)-O(9)-H(9B)	104.3	N(4)-C(5)-H(5)	118.2
Ca(01)-O(7)-H(7A)	126.1	C(4)-C(5)-H(5)	118.2
Ca(01)-O(7)-H(7B)	121.4	C(9)-C(8)-H(8)	120.9
H(7A)-O(7)-H(7B)	104.5	C(7)-C(8)-H(8)	120.9

N(1)-C(6)-H(6)	118.5	C(3)-C(2)-H(2)	120.1
C(7)-C(6)-H(6)	118.5	C(1)-C(2)-H(2)	120.1

Symmetry transformations used to generate equivalent atoms:

#1 x-1,y,z   #2 x+1,y,z

Table S3. Atomic coordinates (× 10^4) and equivalent isotropic displacement parameters ($Å^2$ × 10^3) for CPS. U(eq) is defined as one-third of the trace of the orthogonalized U_{ij} tensor.

Atom	*x*	*y*	*z*	U(eq)
Ca(1)	3903.7(7)	1970.6(5)	7723.7(5)	21(1)
S(2)	8930.3(9)	702.5(6)	8000.0(6)	22(1)
S(1)	6929.4(9)	4844.9(6)	6818.7(6)	25(1)
O(3)	6299(3)	3575.4(17)	6962.2(17)	30(1)
O(4)	7099(2)	962.6(17)	8483.0(16)	27(1)
O(2)	5452(3)	5831.8(17)	6297.1(17)	34(1)
O(5)	10406(2)	1592.3(17)	7985.8(16)	27(1)
O(8)	3569(3)	1967.8(18)	9784.0(17)	26(1)
O(10)	3844(3)	1887(2)	5767(2)	34(1)
O(9)	3585(3)	-330.4(17)	8534.0(17)	34(1)
O(1)	8752(3)	5164.2(19)	6184.5(18)	37(1)
O(6)	9537(3)	-639.4(17)	8629.5(17)	30(1)
O(7)	2336(3)	4034.1(18)	6927(2)	39(1)
N(4)	7386(3)	66(2)	4974(2)	37(1)
N(1)	7057(3)	5796(2)	9819(2)	36(1)
C(6)	7747(4)	4719(3)	10698(3)	37(1)
C(10)	6833(4)	5800(3)	8675(3)	32(1)
C(3)	8914(4)	2092(3)	5465(2)	30(1)
C(9)	7284(4)	4758(2)	8365(2)	26(1)
C(7)	8251(4)	3640(3)	10464(3)	36(1)
C(1)	7744(4)	1199(3)	4061(3)	40(1)
C(4)	8531(3)	936(2)	6421(2)	23(1)
C(5)	7777(4)	-43(3)	6136(3)	32(1)
C(8)	8021(4)	3652(3)	9278(3)	31(1)
C(2)	8501(4)	2213(3)	4267(3)	37(1)
H(8A)	3451	2736	9742	40
H(9A)	2457	-529	8831	51
H(9B)	4317	-676	9163	51
H(7A)	1182	4186	6685	58
H(7B)	2930	4704	6475	58
H(6)	7894	4696	11502	45
H(10)	6348	6542	8056	38
H(3)	9433	2766	5624	36
H(7)	8740	2912	11099	43
H(1)	7467	1305	3245	48

H(5)	7530	-819	6788	38
H(8)	8352	2938	9098	38
H(2)	8733	2980	3599	45
H(8B)	2730(40)	1570(30)	10120(30)	34(10)
H(10A)	3960(50)	2490(30)	5240(30)	42(12)
H(10B)	3430(50)	1350(40)	5550(30)	58(12)

Table S4. The related bond length (Å) and angle (°) of hydrogen bonds in CPS.

D-H···A	d(D-H)/Å	d(H···A)/Å	d(D···A)/Å	D-H···A/°
O9-H9A•••O6#1	0.85	2.07	2.852(3)	152.1
O9-H9B•••O8#2	0.85	2.1	2.892(3)	154.9
O7-H7A•••O1#1	0.85	1.97	2.778(3)	157.8
O10-H10A•••O2#3	0.72(3)	2.09(3)	2.811(3)	175(4)

Symmetry codes:

#1 -1+x,y,z #2 1-x,-y,2-z #3 1-x,1-y,1-z

Table S5. Comparison of optical properties between selected sulfate-related materials.

Crystals	Space Group	Cutoff (nm)	Band gap (eV)	Birefringence
Li_2SO_4[1]	$P2_1/c$	~200	6.04[a]	0.004@546 nm[b]
$LiNH_4SO_4$[2]	$P2_1cn$	171	7.16[a]	0.0078@552 nm[b]
$NaRbY_2(SO_4)_4$[3]	$C2/c$	＜200	5.71[a]	0.045@550 nm[b]
$NaSb_3O_2(SO_4)_3{\cdot}H_2O$[4]	$P\bar{1}$	350	3.91[a]	0.041@1064 nm[a]
$Sn_3O_2(OH)(HSO_4)$[5]	$Pca2_1$	308	3.30[a]	0.169@546 nm[b]
$[C(NH_2)_3]_2S_2O_8$[6]	$P4_12_12$	222	4.25[b]	0.018@546 nm[b]
$K_2S_4O_6$[7]	Cc	298	4.0[b]	0.066@1064 nm[a]
$Sr(NH_2SO_3)_2$[8]	Pc	＜190	7.32[a]	0.027@546.1 nm[b]
$Ba(NH_2SO_3)_2$[8]	$Pna2_1$	＜190	7.29[a]	0.028@546.1 nm[b]
$Ba(SO_3CH_3)_2$[9]	$Cmc2_1$	159	7.8[b]	0.04@589.3 nm[b]
$SO_2(NH_2)_2$[10]	$Fdd2$	160	7.75[b]	0.07@589.3 nm[b]
$HN(SO_2F)_2$[11]	$\mathrm{P2_1}$	149	5.97[a]	0.067@546 nm[a]
$Cs(3\text{-}C_5H_4NSO_3)$[12]	$P2_1/c$	283	4.2[a]	0.266@546 nm[a]
$Ca(3\text{-}pySO_3)_2{\cdot}4H_2O$	$P\bar{1}$	257	4.4[b]	0.286@532 nm[b]

[a]Experimentally measured. [b]Theoretically calculated.

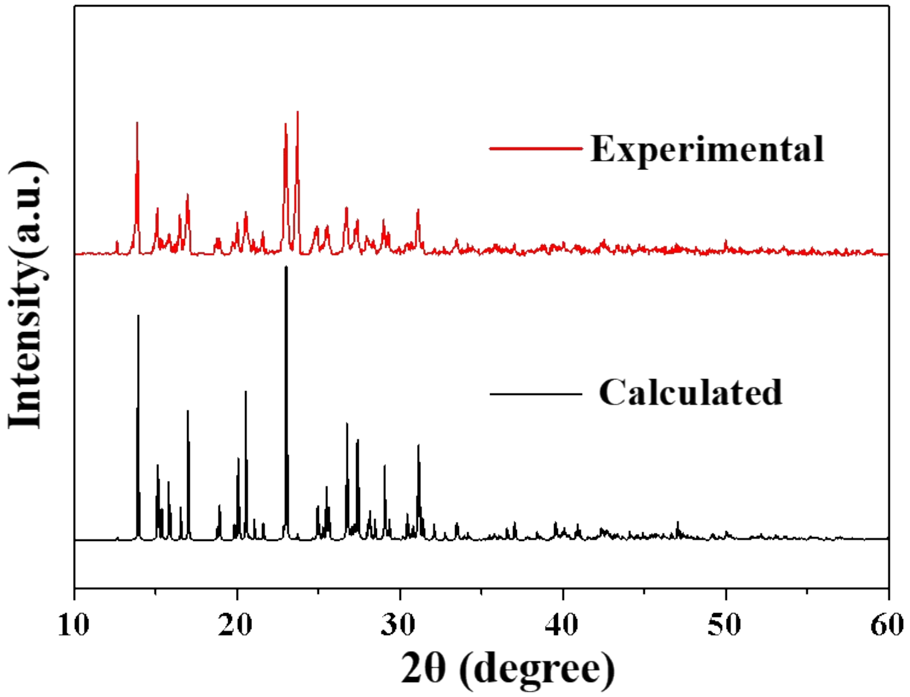

Fig. S1 Experimental and calculated PXRD patterns of CPS.

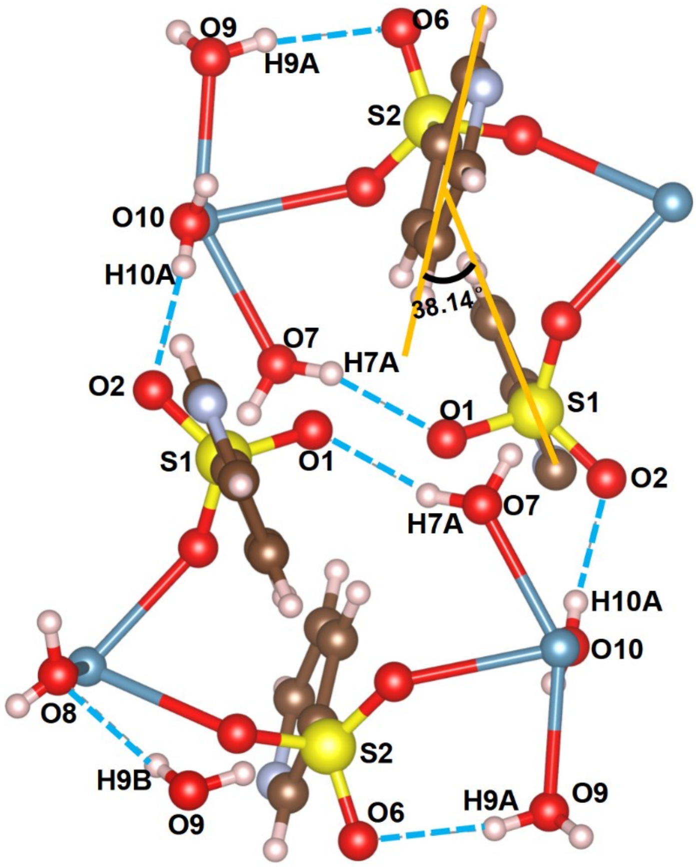

Fig. S2 Types of hydrogen bonds in the structure of CPS and the coplanarity of pyridine rings in [3-pySO_3] groups.

REFERENCES

1 W. Jin, W. Zhang, A. Tudi, L. Wang, X. Zhou, Z. Yang and S. Pan, *Adv. Sci.*, 2021, **8**, 2003594.

2 Y. Song, H. Yu, B. Li, X. Li, Y. Zhou, Y. Li, C. He, G. Zhang, J. Luo and S. Zhao, *Adv. Funct. Mater.*, 2024, **34**, 2310407.

3 Y. Zhao, Y. Song, Y. Li, W. Liu, Y. Zhou, W. Huang, J. Luo, S. Zhao and B. Ahmed, *Inorg. Chem.*, 2024, **63**, 11187–11193.

4 K. Wang, X.-F. Li, C. He, J.-H. Li, X.-T. An, L. Wei, Q. Wei and G.-M. Wang, *Cryst. Growth. Des.*, 2022, **22**, 478–484.

5 Y. Chen, H. Luo, Z. Yin, X. Dong, D. Gao, Y. Zhou, L. Huang, L. Cao and G. Zou, *Inorg. Chem.*, 2024, **63**, 15206–15214.

6 M. Zhang, B. Zhang, D. Yang and Y. Wang, *Inorg. Chem. Front.*, 2022, **9**, 6067–6074.

7 T. Huang, Y. Xiao, J. Gu, Y. Wang, K. Wu and B. Zhang, *J. Mater. Chem. C*, 2022, **10**, 17190–17195.

8 X. Hao, M. Luo, C. Lin, G. Peng, F. Xu and N. Ye, *Angew. Chem., Int. Ed.*, 2021, **60**, 7621–7625.

9 H. Tian, C. Lin, X. Zhao, F. Xu, C. Wang, N. Ye and M. Luo, *CCS Chem.*, 2023, **5**, 2497–2505.

10 H. Tian, N. Ye and M. Luo, *Angew. Chem., Int. Ed.*, 2022, **61**, e202200395.

11 H. Zhou, S. Shu, A. Tudi, W. Jin, S. Pan and Z. Yang, *Adv. Optical Mater.*, 2024, **12**, 2400780.

12 Z. Bai and K. M. Ok, *Angew. Chem., Int. Ed.*, 2024, **63**, e202315311.
